# Microperimetry Sensitivity Correlates to Structural Macular Changes in Adolescents with Achromatopsia Unlike Other Visual Function Tests

**DOI:** 10.3390/jcm13195968

**Published:** 2024-10-08

**Authors:** Eleonora Cosmo, Elisabetta Pilotto, Enrica Convento, Federico Parolini, Edoardo Midena

**Affiliations:** 1 Department of Neuroscience—Ophthalmology, University of Padova, 35128 Padova, Italy; eleonora.cosmo@unipd.it (E.C.); enrica.convento@unipd.it (E.C.); federico.parolini@studenti.unipd.it (F.P.); edoardo.midena@unipd.it (E.M.); 2 IRCCS—Fondazione Bietti, 00198 Rome, Italy

**Keywords:** achromatopsia, retinal inherited disease, cone dystrophy, microperimetry, optical coherence tomography, best corrected visual acuity, low luminance visual acuity, contrast sensitivity

## Abstract

**Objectives:** Achromatopsia (ACHM) is a rare autosomal, recessively inherited disease that is characterized by cone dysfunction, for which several gene therapies are currently on trial. The aim of this study was to find correlations between the morphological macular changes identified using optical coherence tomography (OCT) and some visual functional parameters. Visual acuity (VA), contrast sensitivity (CS), and macular sensitivity obtained by means of microperimetry were assessed. **Methods:** Adolescents with ACHM underwent macular microperimetry (S-MAIA device) in mesopic condition, macular OCT, best corrected visual acuity (BCVA), low luminance visual acuity (LLVA), near vision acuity (NVA), and CS measurement. **Results:** Eight patients (15 eyes) with ACHM were analyzed. The mean age was 17 ± 2.7 years, and genetic variants involved the CNGA3 gene (37.5%) and CNGB3 gene (62.5%). OCT staging significantly correlated with microperimetry sensitivity parameters, namely the sensitivity of the central foveal point (*p* = 0.0286) and of the first and second perifoveal rings (*p* = 0.0008 and *p* = 0.0014, respectively). No correlations were found between OCT staging and VA measurements, nor with CS value. **Conclusions:** Among the extensive evaluated visual function tests, only microperimetry sensitivity showed a correlation with morphological macular changes identified at OCT. Microperimetry sensitivity may thus represent a useful visual function tool in natural ACHM history studies considering the upcoming research on gene therapies for the treatment of ACHM.

## 1. Introduction

Achromatopsia (ACHM) is a rare autosomal, recessively inherited disease that is characterized by cone dysfunction. It affects approximately 1 in 30,000 individuals [1]. Genetic variants responsible for the disease have been identified in 6 genes—CNGA3 and CNGB3 (mutated in approximately 70% of all ACHM cases), GNAT2, PDE6C, PDE6H, and ATF6—all encoding for components of the cone phototransduction cascade with the exception of ATF6, which plays a role in the regulation of the unfolded protein response and endoplasmic reticulum homeostasis [2,3,4]. From early infancy, affected individuals experience a reduction in visual acuity, photophobia, pendular nystagmus of high frequency and low amplitude, reduced or absent color perception, and central scotomata. Ophthalmoscopic fundus appearance may be unremarkable. Some subtle retinal pigment epithelium (RPE) disturbance and/or foveal atrophic changes or a dull foveal reflex might be found in some cases. Structural changes in the central macula are present at optical coherence tomography (OCT) such as variable degrees of foveal hypoplasia, inner segment/outer segment junction (IS/OS) disruption, and outer nuclear layer loss [5,6]. In recent years, multiple studies have focused on assessing the eventual progressive nature of ACHM with conflicting results. Some works concluded with the essential stability of the disease [6,7,8], whereas the majority of the studies identified slow and subtle alterations in retinal structure and visual function [5,9,10,11,12,13,14,15,16,17]. Knowledge of ACHM natural history is essential considering the recent gene therapy approach. Gene therapy in relation to ACHM has recently gone from animal models to phase I/II trials in humans. Mild visual functional improvement consisting of a modest increase in visual acuity and contrast sensitivity, as well as favorable changes in color vision, photoaversion, and vision-related quality of life questionnaire scores, were found [18,19,20,21,22]. However, a consensus on a visual function endpoint when treating ACHM patients is still to be defined. Thus, considering the forthcoming gene therapy, it becomes fundamental to evaluate which functional test may represent the optimal functional endpoint in achromatic populations. In addition to the more commonly used visual acuity measurement, macular sensitivity by means of microperimetry may acquire clinical importance. Only a few studies have focused on macular sensitivity changes using microperimetry in patients with ACHM [6,7,8,14,23,24]. A progressive decrease in macular sensitivity or a negative correlation of macular sensitivity with patients’ age has been reported [6,14]. Other studies did not confirm such a decline [7,8,24]. Sundaram et al. found no correlations between mean microperimetry sensitivity and retinal morphology at OCT in a large population of ACHM patients, but their cohort included patients with an extensive age range [6].

The present study aimed to investigate the correlations among morphological changes assessed with OCT and extensive functional parameters such as best corrected visual acuity (BCVA), low luminance visual acuity (LLVA), contrast sensitivity (CS), and macular microperimetry sensitivity in a cohort of ACHM adolescents.

## 2. Materials and Methods

### 2.1. Population

We conducted an observational cross-sectional study. Adolescents affected by ACHM that were followed at our pediatric low vision unit were consecutively enrolled. The study was conducted according to the guidelines of the Declaration of Helsinki and was approved by the local Ethics Committee (approval code 73/n/AO/20; approval date 1 January 2021). Informed consent was obtained from all the enrolled subjects or their legal guardian for patients < 18 years old. The inclusion criterion was genetically confirmed achromatopsia.

### 2.2. Visual Acuity and Contrast Sensitivity

All patients underwent a complete ophthalmological evaluation including best corrected visual acuity (BCVA) and low luminance visual acuity (LLVA) measurements using Early Treatment Diabetic Retinopathy Study (ETDRS) charts, according to a standard protocol, which was previously described in [25], as well as near vision acuity (NVA) evaluation using MNREAD charts and contrast sensitivity (CS) evaluation using Pelli–Robson charts. BCVA, LLVA, and NVA were expressed in logMAR notation and CS was expressed in log units.

### 2.3. Microperimetry

**Figure 1:**
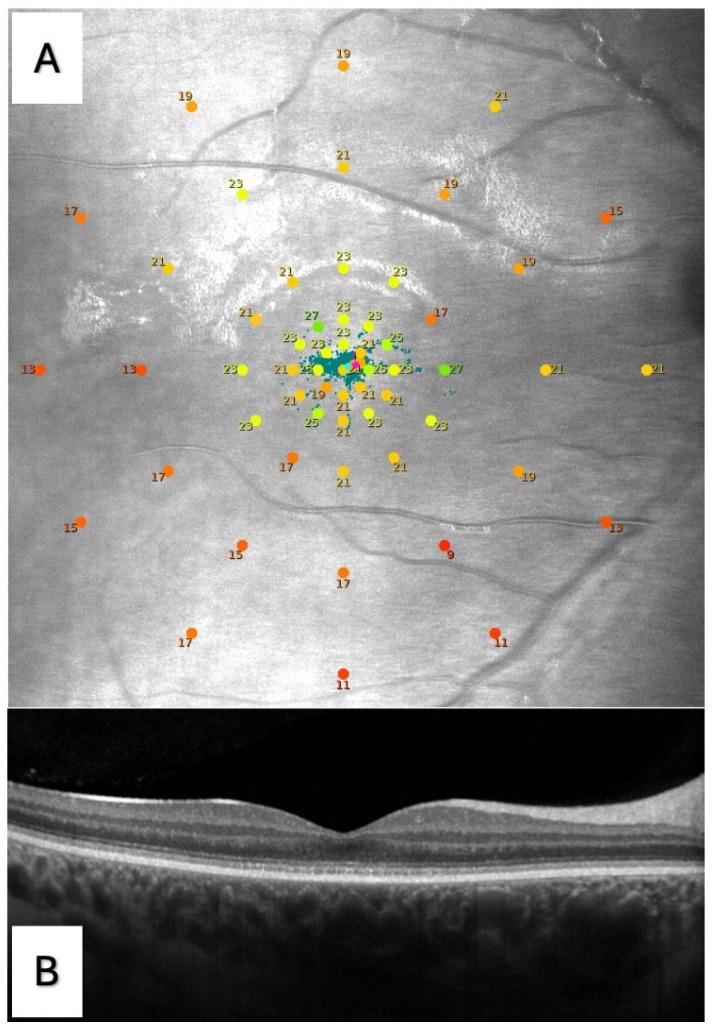
(**A**) Microperimetry of the right eye of a patient with achromatopsia (ACHM) performed with the S-MAIA device (CenterVue Spa, Padova, Italy). A customized grid with 57 points centered on the fovea was used, with the following distribution: one central point; 8 points in a first ring at 1° from the center (R1); and 12 points in each of the other four rings localized, respectively, at 2° (R2), 4° (R3), 8° (R4), and 12° (R5) from the center. The sensitivity of the single tested points is expressed in decibel (dB), ranging from 0 to 36 dB, with the 0 dB stimulus of maximum intensity. Microperimetry color guide—red from 0 to 23 dB, yellow from 23 to 25 dB, and green from 25 to 36 dB. (**B**) Linear B-scan optical coherence tomography (OCT) of the same eye with stage 1, according to the OCT staging proposed by Greenberg et al. [11].

Macular mesopic microperimetry was performed using the S-MAIA device (Scotopic Macular Integrity Assessment, CenterVue Spa, Padova, Italy) after pupil dilation with one eye drop of Tropicamide 1%. Both eyes of the patients were analyzed. The test was taken under mesopic conditions with a white stimulus on a white background. Testing parameters included a Goldmann size III stimulus, a 4-2 strategy threshold, and a customized macular grid with 57 points centered on the fovea and distributed as follows: a central point (C); 8 points in a first ring at 1° from the center (R1); and 12 points in each of the other four rings localized, respectively, at 2° (R2), 4° (R3), 8° (R4), and 12° (R5) from the center (Figure 1). Such a pattern was designed in order to provide a fairly regular sampling density of retinal sensitivity of the macular region, with a higher density in the fovea. Dynamic macular fixation was evaluated using the same device while performing the microperimetry sensitivity test, and was expressed by means of the bivariate contour ellipse area (BCEA), which is the area of an ellipse comprising 95% (BCEA95) and 63% (BCEA63) of fixation points depending on the standard deviations of the horizontal and vertical eye positions recorded while the patient is fixating [26].

The following microperimetry parameters were collected: BCEA63, BCEA95, average sensitivity (AS), central point sensitivity (C), and average sensitivity of each of the five concentrical rings of the macular grid (R1, R2, R3, R4, and R5).

### 2.4. Optical Coherence Tomography

All patients underwent optical coherence tomography (OCT) with Spectralis HRA + OCT (Heidelberg Engineering, Heidelberg, Germany). Linear scans passing through the fovea were acquired for each eye (Figure 1). The best quality image (consistently with the nystagmus characterizing the disease) was chosen for analyzing the morphological alterations of the outer retina and retinal pigment epithelium (RPE) layer. Each eye was then categorized into 5 stages, according to a previously proposed staging [11], as follows: stage 1 was characterized by an intact outer retina, even with possible subtle discontinuities of the inner segment ellipsoid (ISe) line; stage 2 was defined as the disruption of the ISe line but without the presence of an optically empty space (OES); stage 3 demonstrated an OES with the loss of photoreceptor inner and outer segments, but without visible RPE damage; stage 4 was characterized by the presence of an OES with partial RPE disruption; and stage 5 was defined as complete RPE disruption with or without the loss of the outer nuclear layer (ONL). Two blinded independent expert ophthalmologists (EC and EP) graded each eye, with complete agreement.

### 2.5. Statistical Analysis

Investigated parameters were summarized by means of common elements of descriptive statistics, namely mean, standard deviation, minimum and maximum range for quantitative variables, and absolute and relative frequency in percentages for qualitative variables. A linear regression model, adjusted for the replication of measurements when both eyes of the patients were included, was used to search for the correlations between OCT stage and visual function tests and among all visual function parameters. For all the analyses, SAS^®^ v.9.4 (SAS Institute, Cary, NC, USA) was used. All tests have been interpreted as statistically significant if *p* < 0.05.

## 3. Results

### 3.1. Population

Our study involved 8 patients affected by ACHM—2 (25%) males and 6 (75%) females. The mean age was 17 ± 2.7 years (range 14–23 years old). Five patients (62.5%) carried mutations in the CNGB3 gene and three patients (37.5%) in the CNGA3 gene. Both eyes of each patient contributed to morphological and functional evaluation, with the exception of one eye, which was unable to undergo microperimetry due to poor BCVA (1.4 logMAR). Thus, clinical evaluations and the following analysis included 15 eyes of 8 patients. According to morphological OCT changes, 5 eyes (33.2%) had stage 1, 6 eyes (40%) had stage 2, 0 eyes (0%) had stage 3, 2 eyes (13.3%) had stage 4, and 2 eyes (13.3%) had stage 5. Data on demographic features, genotype, and OCT staging are reported in Table 1.

Mean BCVA, LLVA, NVA, and CS were 0.7 ± 0.3 logMAR, 0.8 ± 0.3 logMAR, 0.3 ± 0.2 logMAR, and 1.4 ± 0.2 logCS, respectively. The average macular sensitivity (AS) and central point sensitivity were 21.2 ± 2.7 dB and 20.4 ± 6.6 dB, respectively. Functional data including all microperimetry parameters are reported in Table 2.

### 3.2. Morpho-Functional Correlations

The central foveal sensitivity (C) and mean sensitivity of R1 and R2 significantly correlated with OCT staging (*p* = 0.0286, *p* = 0.0008, and *p* = 0.0014, respectively). BCEA63 and BCEA93 did not correlate with OCT staging (*p* > 0.05 for all).

None of the visual acuity measurements (BCVA, LLVA, and NVA) and contrast sensitivity measurements were statistically associated with OCT staging (*p* > 0.05 for all).

All morpho-functional correlations are reported in Table 3.

### 3.3. Visual Functional Parameter Correlations

BCEA63 and BCEA95 positively correlated with LLVA with statistical significance (*p* < 0.0001 for both), and with BCVA with a borderline significance (*p* = 0.0508 and *p* = 0.0507, respectively).

The microperimetry sensitivity of the central foveal sensitivity (C) of R1 and R2 negatively correlated with NVA, significantly for R1 and R2 (*p* = 0.0033 and *p* = 0.0025, respectively), and with a borderline significance for C (*p* = 0.0521). The sensitivity of R5 positively correlated with BCVA (*p* = 0.0295). The correlations among all visual functional parameters are reported in Table 4.

## 4. Discussion

ACHM is a rare autosomal recessive disease characterized by cone dysfunction. To date, six genes have been identified as responsible for the disease, i.e., CNGA3, CNGB3, GNAT2, PDE6C, PDE6H, and ATF6. Among them, CNGA3 and CNGB3, encoding, respectively, for the α- and β-subunits of the cone photoreceptor cyclic nucleotide-gated ion channel, account for approximately 70% of all cases. For this reason, ACHM represents an attractive candidate for gene therapy. Recently, several small-scale studies preliminarily assessed the safety and potential efficacy of subretinal gene therapy in ACHM associated with CNGA3 and CNGB3 [19,20,21,22,27]. No correlation of retinal morphology with visual acuity, contrast sensitivity, or color confusion index have been detected until now [6,8]. Objectively measurable clinical visual functional outcomes that may be correlated to OCT changes must still be defined to evaluate the efficacy of the upcoming gene therapies.

The main finding of our study was that only microperimetry macular sensitivity correlated with OCT staging. All the other functional tests, including BCVA, LLVA, NVA, and CS, did not. In particular, the sensitivity of the first and second concentrical ring, corresponding to the fovea and perifoveal area (1- and 2-degree radius, respectively) correlated to OCT structural changes. Previously, only Sundaram et al. tested the correlation between microperimetry sensitivity and OCT staging in ACHM, and no clear morpho-functional associations were detected [6]. However, they only analyzed the mean macular sensitivity of an area of 8-degree radius, using a macular grid of 44 tested points. In our approach, we used a customized macular grid with a higher number of tested points (57) that were distributed with a higher density in the fovea and perifoveal area. Furthermore, in addition to the mean sensitivity of the whole macular tested area, we also separately analyzed the sensitivity of the central foveal point and the mean sensitivity of each of the five concentrical rings of the grid. A strong correlation with OCT staging was only present for the sensitivity of the central point and for the mean sensitivity of the first and second ring, which extended up to 2-degree radius from the foveal center. Such results are consistent with the peculiar morphological retinal changes limited to the fovea and perifoveal region, where cone photoreceptors are packed [28]. Our results support microperimetry sensitivity as a useful functional parameter in patients with ACHM as it objectively correlates with OCT changes. Visual acuity (BCVA, LLVA, and NVA), contrast sensitivity, and fixation area failed to correlate with OCT staging. Such findings mirror what has already been observed in other macular diseases when involving the fovea, such as age-related macular degeneration (AMD), particularly when geographic atrophy (GA) areas progress towards the fovea [29,30]. Some functional parameters such as visual acuity and fixation turned out to be dependent not only according to disease progression, but also according to vision experience and bilateral disease. Visual acuity and fixation are capable of being trained and improving, regardless of macular changes [31,32]. Microperimetry can objectively quantify macular function independently of the aforementioned factors and in a topographic-related manner. Using microperimetry, macular sensitivity is investigated in close relation to macular morphology detectable at structural OCT. Microperimetry sensitivity is unrelated to vision experience and to fellow eye morphological and functional conditions. Microperimetry sensitivity has been recently accepted as a useful functional endpoint tool in clinical trials investigating macular teleangectasia (MacTel) type 2 [33].

When analyzing correlations between microperimetry parameters and the other functional data, fixation stability directly and significantly correlates with LLVA, while the direct correlation with BCVA showed a borderline significance. In progressive macular diseases with foveal threat, such as AMD or Stargardt disease type 1 (STGD1), fixation stability is strictly related to BCVA [34,35]. In ACHM, the stronger correlation with LLVA rather than with BCVA is probably related to the photophobia that characterizes patients with ACHM. Therefore, assessing visual acuity at decreased levels of luminance, as in LLVA measurements, may limit the influence of photoaversion on visual acuity evaluation in patients with ACHM. Our results highlight the utility of LLVA as a possible surrogate marker for VA evaluation in patients with ACHM. In our view, this should be taken into account in the design of future clinical trials. Finally, we identified a direct correlation between near vision acuity and the microperimetry sensitivity of the central point and of the first and second concentrical rings, underlining how the reduction in retinal sensitivity in the central area impacts visual performance in patients with ACHM.

The main limitation of this study is the reduced sample size, but this is a common issue in studies on rare eye diseases, when limited to young subjects. Such a limitation may have affected the statistical power of the results and the lack of statistical significance in some tested morpho-functional correlations. The resulting statistically significant association between OCT staging and macular sensitivity, even in such a small cohort of patients, is of clinical relevance. Studies with a larger population will be necessary to confirm our results. We included a cohort of adolescents with ACHM, homogeneous in age and genetic variants. ACHM in our patients was linked to either CNGA3 (37.5%) or CNGB3 (62.5%). Another limitation of this study is its cross-sectional design. Longitudinal studies are needed to fully understand the progression of achromatopsia and validate our findings. Future studies will have to focus on a follow-up longitudinal design and possibly a wider population. These studies may lead to other morpho-functional correlations during the progression of the disease.

In conclusion, in adolescents with ACHM, among all the visual function tests, only microperimetry sensitivity correlates with the morphological macular changes identified at OCT. Such a correlation is evident when considering the sensitivities of the fovea and perifoveal area; thus, a customized microperimetry macular grid is essential when evaluating patients with ACHM. Microperimetry sensitivity in ACHM is, in our view, an objective visual function outcome and therefore deserves further investigation considering the advent of new gene therapeutic strategies targeting ACHM.

## Figures and Tables

**Table 1:** Demographic features, genetic mutations, and OCT staging of the study population.

Demographic Features (8 Subjects)	
Sex, M:F (N, %)	2, 25:6, 75
Age, years (mean ± SD)	17 ± 2.7
Genetic mutation (8 subjects)	(N, %)
CNGA3	3, 37.5
CNGB3	5, 62.5
OCT stage (15 eyes)	(N, %)
Stage 1	5, 33.2
Stage 2	6, 40
Stage 3	0, 0
Stage 4	2, 13.3
Stage 5	2, 13.3

M: male; F: female; N: number, SD: standard deviation; OCT: optical coherence tomography.

**Table 2:** Functional parameters tested in the study population.

Functional Parameter	Mean ± SD
BCVA, logMAR	0.7 ± 0.3
LLVA, logMAR	0.8 ± 0.3
NVA, logMAR	0.3 ± 0.2
CS, logCS	1.4 ± 0.2
Microperimetry	
BCEA63, degree2	13.0 ± 16.0
BCEA95, degree2	39.0 ± 47.9
AS, dB	21.2 ± 2.7
C, dB	20.4 ± 6.6
R1, dB	20.8 ± 5.9
R2, dB	21.1 ± 4.7
R3, dB	23.1 ± 2.0
R4, dB	21.5 ± 2.9
R5, dB	20.2 ± 2.7

VA: visual acuity; CS: contrast sensitivity; BCVA: best corrected visual acuity; LLVA: low luminance visual acuity; NVA: near vision acuity; BCEA: bivariate contour ellipse area; SD: standard deviation; AS: average sensitivity; dB: decibel; C: central foveal point sensitivity; R1, R2, R3, R4, R5: ring of sensitivity, respectively, at 1°, 2°, 4°, 8°, and 12° from the center.

**Table 3:** Correlations between functional parameters and OCT staging.

Functional Parameters	OCT Staging (Mean ± SD)				*p*-Value
	1 (*n* = 5)	2 (*n* = 6)	4 (*n* = 2)	5 (*n* = 2)	
BCEA63, degree^2^	18.7 ± 27.0	6.7 ± 6.6	17.0 ± 3.5	13.9 ± 1.4	0.8098
BCEA95, degree^2^	56.0 ± 80.8	20.1 ± 19.7	51.1 ± 10.7	41.7 ± 4.3	0.8096
AS, dB	21.9 ± 2.1	22.5 ± 1.4	23.9 ± 0.9	17.0 ± 3.1	0.0694
C, dB	21.1 ± 1.8	23.3 ± 2.4	24.0 ± 1.4	8.5 ± 12.0	0.0286
R1, dB	21.9 ± 2.1	23.5 ± 1.0	23.4 ± 0.5	7.2 ± 5.7	0.0008
R2, dB	21.1 ± 1.8	23.9 ± 0.9	23.3 ± 0.5	10.5 ± 4.2	0.0014
R3, dB	21.8 ± 1.7	23.4 ± 1.1	25.5 ± 0.9	22.8 ± 4.1	0.1682
R4, dB	19.7 ± 2.9	21.7 ± 2.8	24.8 ± 1.4	22.3 ± 1.6	0.5558
R5, dB	18.7 ± 3.5	20.4 ± 2.4	22.4 ± 1.3	21.3 ± 1.2	0.5336
BCVA, logMAR	0.620 ± 0.390	0.633 ± 0.350	0.700 ± 0.000	0.800 ± 0.000	0.9865
LLVA, logMAR	0.820 ± 0.492	0.700 ± 0.253	0.700 ± 0.000	0.800 ± 0.000	0.9181
NVA, logMAR	0.280 ± 0.164	0.217 ± 0.098	0.200 ± 0.000	0.600 ± 0.000	0.2608
CS, logCS	1.260 ± 0.272	1.400 ± 0.122	1.425 ± 0.106	1.575 ± 0.106	0.6628

OCT: optical coherence tomography; SD: standard deviation; *n*: number; BCEA: bivariate contour ellipse area; AS: average sensitivity; dB: decibel; C: central foveal point sensitivity; R1, R2, R3, R4, R5: ring of sensitivity, respectively, at 1°, 2°, 4°, 8°, and 12° from the center; BCVA: best corrected visual acuity; LLVA: low luminance visual acuity; NVA: near vision acuity; CS: contrast sensitivity.

**Table 4:** *p*-values of correlations between microperimetry parameters and BCVA, LLVA, NVA, and CS.

Microperimetry Parameter	BCVA	LLVA	NVA	CS
BCEA63	0.0508 (B) *	<0.0001 *	0.2750	0.9941
BCEA95	0.0507 (B) *	<0.0001 *	0.2753	0.9427
AS	0.5096	0.5124	0.0797	0.7608
C	0.6194	0.5462	0.0521 (B) †	0.2888
R1	0.7506	0.9414	0.0033 †	0.1758
R2	0.7914	0.9823	0.0025 †	0.3006
R3	0.3578	0.4930	0.9451	0.2175
R4	0.0724	0.1680	0.6010	0.2199
R5	0.0295 *	0.0618	0.2755	0.6525

BCVA: best corrected visual acuity; LLVA: low luminance visual acuity; NVA: near vision acuity; CS: contrast sensitivity; BCEA: bivariate contour ellipse area; AS: average sensitivity; C: central foveal point sensitivity; R1, R2, R3, R4, R5: ring of sensitivity, respectively, at 1°, 2°, 4°, 8°, and 12° from the center. B indicates borderline significance. *: positive correlation; †: negative correlation.

## Data Availability

The data presented in this study are available in the article. Eventual additional data are available on request from the corresponding author.
